# miR-106b suppresses pathological retinal angiogenesis

**DOI:** 10.18632/aging.202404

**Published:** 2020-12-23

**Authors:** Catherine Ménard, Ariel M. Wilson, Agnieszka Dejda, Khalil Miloudi, François Binet, Sergio Crespo-Garcia, Célia Parinot, Frédérique Pilon, Rachel Juneau, Elisabeth MMA Andriessen, Gaëlle Mawambo, John Paul SanGiovanni, Vincent De Guire, Przemyslaw Sapieha

**Affiliations:** 1 Department of Biochemistry, Maisonneuve-Rosemont Hospital Research Centre, University of Montreal, Montreal H1T 2M4, Quebec, Canada; 2 Department of Ophthalmology, Maisonneuve-Rosemont Hospital Research Centre, University of Montreal, Montreal H1T 2M4, Quebec, Canada; 3 Department of Neurology-Neurosurgery, McGill University, Montreal H3A 2B4, Quebec, Canada; 4 Department of Nutritional Sciences, University of Arizona, Tucson, AZ 85719, USA

**Keywords:** age related macular degeneration, miR-106b, PERK, choroidal neovascularization, angiogenesis

## Abstract

MicroRNAs are small non-coding RNAs that post-transcriptionally regulate gene expression. We recently demonstrated that levels of miR-106b were significantly decreased in the vitreous and plasma of patients with neovascular age-related macular degeneration (AMD). Here we show that expression of the miR-106b-25 cluster is negatively regulated by the unfolded protein response pathway of protein kinase RNA-like ER kinase (PERK) in a mouse model of neovascular AMD. A reduction in levels of miR-106b triggers vascular growth both *in vivo* and *in vitro* by inducing production of pro-angiogenic factors. We demonstrate that therapeutic delivery of miR-106b to the retina with lentiviral vectors protects against aberrant retinal angiogenesis in two distinct mouse models of pathological retinal neovascularization. Results from this study suggest that miRNAs such as miR-106b have the potential to be used as multitarget therapeutics for conditions characterized by pathological retinal angiogenesis.

## INTRODUCTION

Age-related macular degeneration (AMD) is a common [1] and complex [2, 3] disease of aging and the leading cause of irreversible loss of sight in elderly people [4–6]. Early forms of AMD are characterized by subretinal lipoproteinaceous deposits, local attrition of photoreceptors [7] and loss of visual sensitivity [8]. Late forms of AMD are defined by geographic atrophy (loss of retinal pigment epithelium and photoreceptors) [9] and/or pathologic choroidal neovascularization (CNV) characterized by vascular sprouting from the choriocapillaris into the neural retina or subretinal space [10]. The neovascular form (NV AMD or exudative AMD) accounts for over 80% of the vision loss associated with AMD [11].

The advent of therapies targeting vascular endothelial growth factor (VEGF) has significantly improved the quality of life of patients suffering from NV AMD [12–15]. However, not all patients with AMD respond to anti-VEGF therapies [16]. Sustained reduction in retinal VEGF levels can lead to neurotoxicity [17] and degeneration of RPE-choriocapillaris in mouse models [18]. Importantly, assessment by fundus photography and fundus fluorescein angiography of patients on anti-VEGF therapy showed accelerated development of geographic atrophy [19, 20]. These findings justify the need for continued exploration of novel therapeutic interventions.

Given that several inflammatory and growth factors in addition to VEGF [10, 21, 22] are associated with the pathogenesis of NV AMD, a multi-targeted approach is warranted. In this regard, therapeutic delivery of miRNAs may offer a promising avenue. miRNAs are small non-coding RNAs of around 20 nucleotides that act as post-transcriptional regulatory elements of most cellular processes [23]. miRNAs mediate repression of gene expression with the potential of a single miRNA to target mRNA transcripts from hundreds of genes [24]. We previously elucidated a specific miRNA signature in the vitreous and plasma of patients with NV AMD and observed a disease-associated increase in miR-146a and a decrease in miR-106b and miR-152 [25]. Interestingly, within our cohort, we found that both vitreous- and plasma-based miR-146a/miR-106b ratios had greater than 90% discriminatory power for classification of patients with NV AMD with an area under the receiver operating characteristic curve of 0,977 in vitreous humour and 0,915 in plasma, suggesting potential for a blood-based diagnostic. These results are concordant with the evidence based in humans and model systems where upregulation of miR-146a, miR-17 (a miR containing the same seed sequence as miR-106b), miR-125 and miR-155 are associated with human AMD and a mouse model of oxygen induced retinopathy (OIR) [26–30]. miRNAs targeting VEGFA (miR-184, miR-150 and miR-106b) have also been found to be downregulated in human AMD and in animal models [25, 28, 31]. Here, we aimed to determine the mechanism leading to the downregulation of mir-106b in AMD and to characterize the therapeutic potential of upregulating miR-106b for NV AMD.

## RESULTS

### miR-106b is downregulated in the choroid after laser-induced CNV

**Figure 1:**
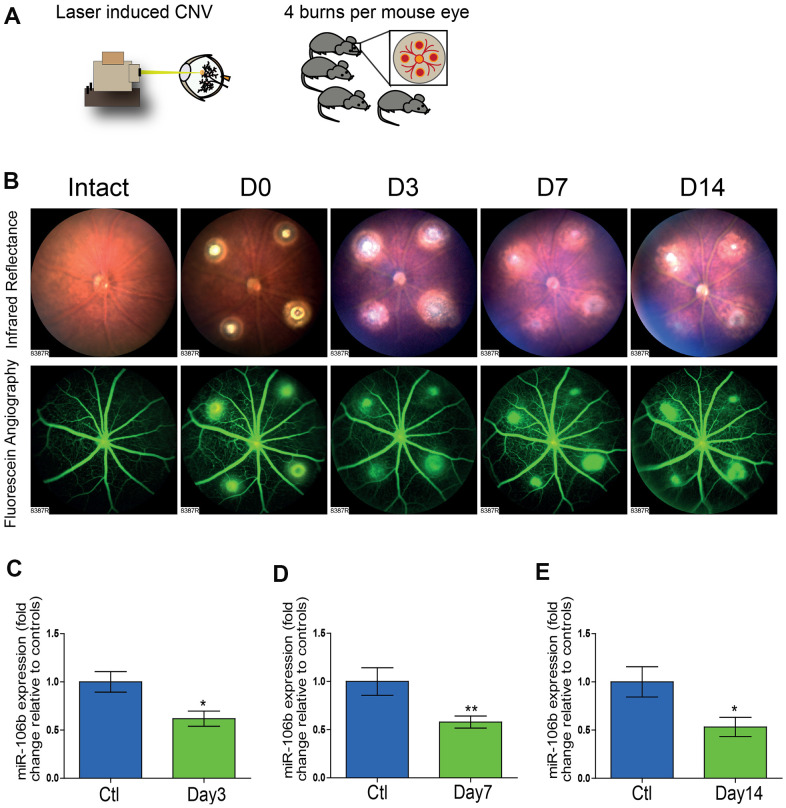
**The initiation of retinal neo-vascularization is associated with downregulation of miR-106b expression.** (**A**) Schematic of laser burn CNV mouse model. (**B**) Representative retinal fundus of a mouse before and after undergoing laser-induced choroidal neovascularization. Serial images depict the progression of neovascularization over a time-course of 14 days in the same eye. Choroidal expression of miR-106b by qPCR relative to intact controls assessed (**C**) 3 days after burns (n=8), (**D**) 7 days after burns (n=13) and (**E**) 14 days after burns (n=10); Scale bar = 50μm. Data are expressed as the mean ± S.E.M. Unpaired Two-tailed Student’s t-test was used for the analysis, *P <0.05; **P<0.001.

In order to evaluate the potential role of miR-106b in CNV and NV AMD, we employed a laser burn-induced neovascularization mouse model. In this model, Bruch’s membrane is ruptured using an argon laser, initiating sprouting of subretinal blood vessels from the choroid, mimicking NV AMD (Figure 1A) [32]. This model is characterized by a reproducible pattern of reduced neovascularization 3 days post-burn, followed by a significant increase in neovascularization that peaks on the 7^th^ day, then vascular regression and wound healing by 14 days post-burn [32]. To illustrate the vascular changes occurring in this model, we took serial ocular fundus images of infrared reflectance and fluorescein angiography after laser burn and subcutaneous injection of fluorescein, revealing fluorescein leakage surrounding burn sites that regressed over time (Figure 1B). In line with our previously reported findings for human vitreous and plasma from patients with active NV AMD [25], choroidal miR-106b expression was significantly downregulated in retinal specimens at all investigated time points, with statistically significant decreases of ~40% at day 3, and ~50% at days 7 and 14, relative to control animals (Figure 1C–1E). The significant downregulation of miR-106b three days post-burn corresponds to the neovascularization nadir in the laser burn model. This suggests that the observed decrease in miR-106b directly precedes initiation of CNV.

### The PERK arm of the unfolded protein response is involved in retinal suppression of the mmu-miR-106b~25 cluster in laser-induced CNV

**Figure 2:**
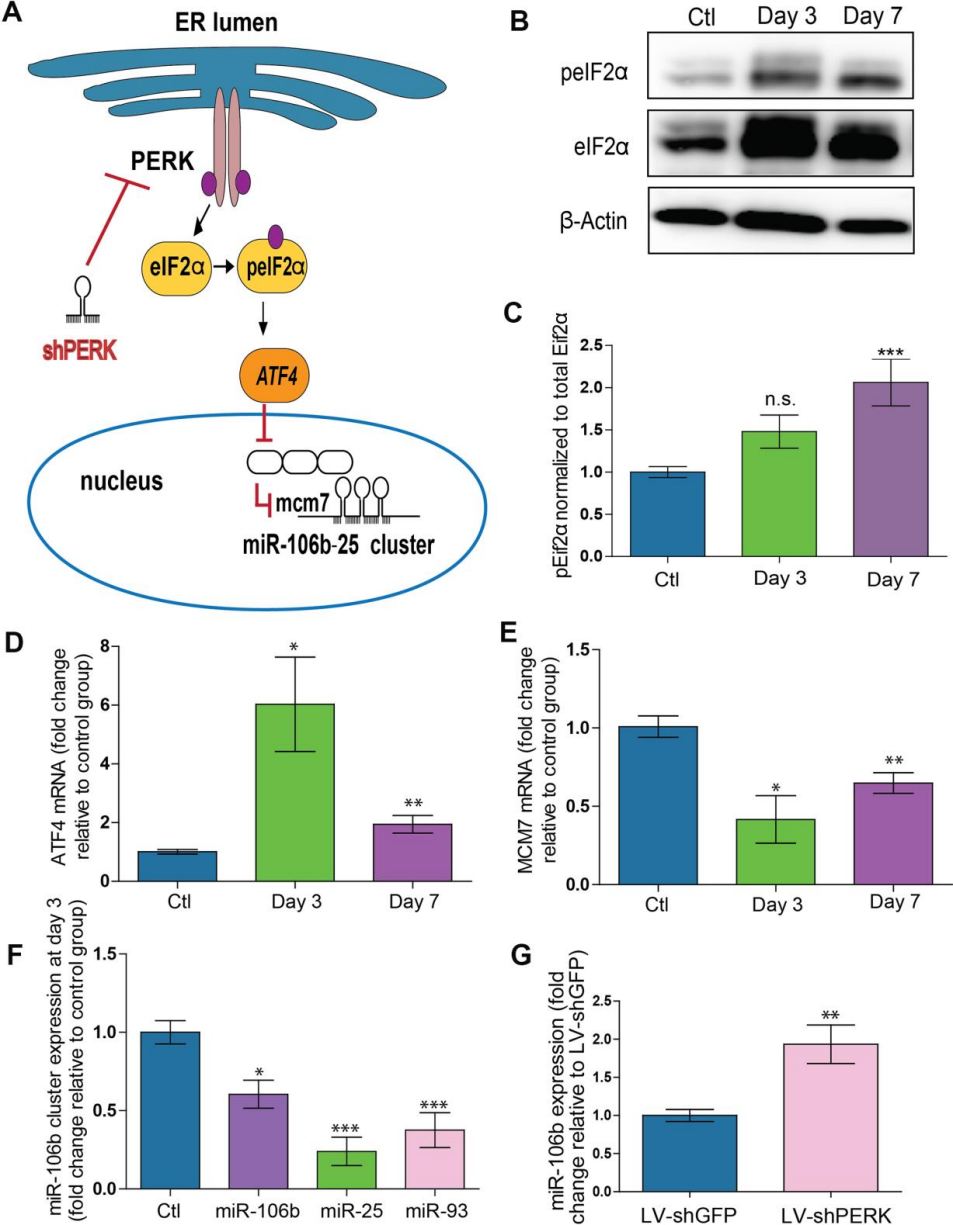
**PERK activation provokes downregulation of miR-106b-25 cluster and MCM7 host gene in a mouse model of CNV.** (**A**) Schematic of ER stress activation of PERK pathway. (**B**) Western blot of Phospho-EIF2α, total-EIF2α and β-actin in control choroids, and 3 and 7 days after burns. (**C**) Western blot quantification in control choroids, 3 days (n=7) and 7 days after burns (n=6). (**D**) *Aft4* mRNA expression in control choroids, 3 days (n=6) and 7 days after burns (n=10). (**E**) *Mcm7* mRNA expression in control choroids, 3 days (n=6) and 7 days after laser burn (n=14). (**F**) miR106~25 cluster member expression in choroids 3 days after laser burn (miR-106b (n=7), miR-25 (n=6) and miR-93 (n=5). (**G**) miR-106b expression after infection of HRMECs with LV.shGFP (negative control), and LV.shPERK (n=5). Data are expressed as mean ± S.E.M. One-way ANOVA with Bonferroni post-hoc test was performed on groups of 3 or more, and unpaired Two-tailed Student’s t-test was used for the analysis of groups of 2, *P <0.05; **P<0.001; ***P<0.0001.

Upon confirmation of a reduction in retinal miR-106b in the CNV mouse model, we next sought to investigate the underlying mechanism. MiR-106b is a member of the miR-106b~25 cluster (mmu-miR-106b, mmu-miR-25 and mmu-miR-93) and is located in the 13^th^ intron of protein-coding gene minichromosome maintenance complex component 7 (*MCM7*) [33]. Regulation of miR-106b expression is tightly correlated with MCM7 transcription. Previous studies have suggested that activation of the protein kinase RNA-like ER kinase (PERK) arm of the UPR and consequent triggering of activating transcription factor 4 (ATF4) as a potential mechanism causing downregulation of the *MCM7* gene and the mmu-miR-106b~25 cluster [34] (Figure 2A). We therefore investigated if the regulation of mmu-miR-106b occurred at the transcriptional level through activation of ER stress effector pathway PERK.

To better characterize PERK activation, we examined its downstream effectors. Phosphorylation of eIF2α was increased after laser burn as was total eIF2α with a significant ~2-fold rise in peIF2α at 7 days (Figure 2B, 2C). Moreover, a significant upregulation of the ATF4 transcript was also detected with an increase at both 3 and 7 days after laser burn (Figure 2D). We then assessed the effect of PERK activation on the *MCM7* gene transcript and miR-106b~25 cluster by qPCR analysis. We observed significant decreases of ~50% and ~35% in *MCM7* transcript expression at 3 and 7 days after laser burn respectively (Figure 2E)**.** Additionally, all members of the miR-106b~25 cluster (miR-106b, miR-25 and miR-93) were downregulated 3 days after laser burn with decreases of ~0.24-fold, ~0.37-fold and ~0.60-fold for miR-25, miR-93 and miR-106b, respectively (Figure 2F). To confirm that PERK can mediate repression of hsa-miR-106b, we infected Human Retinal Microvascular Endothelial Cells (HRMECs) with a lentivirus carrying short hairpin (sh) RNA against PERK. After 72 hours, we observed a significant ~2-fold increase in hsa-miR-106b expression in cultured cells (Figure 2G). Taken together, these results support a PERK-associated decrease in miR-106b in laser-induced CNV.

### miR-106b targets effectors of angiogenesis

**Figure 3:**
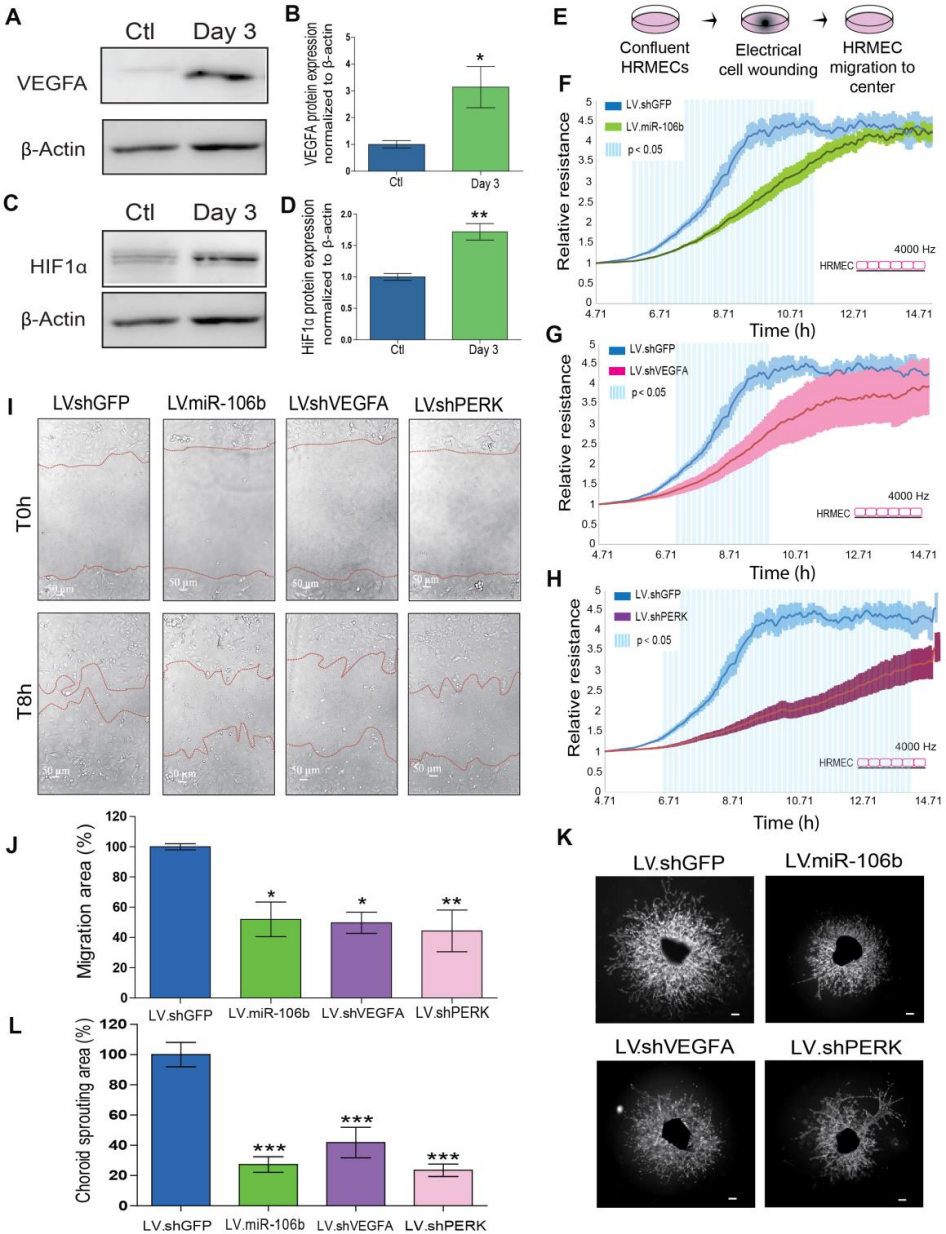
**miR-106b exerts an anti-angiogenic effect and impairs retinal endothelial cell migration.** (**A**) Western blot of VEGFA and β-actin from control choroids and 3 days after burns, and (**B**) quantification (n=4). (**C**) Western blot of HIF1α and β-actin from control choroids and 3 days after burns, and (**D**) quantification (n=4). (**E**) Schematic of ECIS cell migration assay procedure. HRMEC ECIS with (**F**) LV.miR-106b (n=4), (**G**) LV.shVEGFA (n=4) and (**H**) LV.shPERK (n=4) compared to LV.shGFP control. (**I**) HRMEC scratch assay infected 72h with LV.shGFP, LV.miR-106b, LV.shVEGFA and LV.shPERK at T0h and after 8h. (**J**) Migration area quantification of scratch assay with LV.miR-106b (n=4), LV.shVEGFA (n=4), and LV.shPERK (n=4) compared to LV.shGFP. (**K**) Sprouting assay with choroid explants infected with LV.shGFP, LV.miR-106b, LV.shVEGFA, and LV.shPERK. (**L**) Sprouting area quantification with LV.miR-106b (n=9), LV.shVEGFA (n=9), LV.shPERK (n=8) compared to LV.shGFP control. Scale bar = 500 μm. Data are expressed as mean ± S.E.M. Unpaired Two-tailed Student’s t-test was used for the analysis of groups of 2, and one-way ANOVA with Bonferroni post-hoc test was performed on groups of 3 or more, *P <0.05; **P<0.001; ***P<0.0001.

To confirm the involvement of miR-106b in the regulation of AMD-related angiogenesis, we quantified protein levels of experimentally validated targets of miR-106b involved in the neovascularization process. Previous studies demonstrated that miR-106b can influence expression of VEGFA and HIF1α [31]. In line with a decrease in miR-106b, we confirmed the upregulation of VEGFA (~3-fold) and HIF1α (~1.71-fold) in choroid specimens 3 days after laser burn (Figure 3A–3D). Importantly, we did not observe variations at the mRNA level for these targets, suggesting post-transcriptional regulation of their protein expression or altered translation efficiency, characteristic of miRNA regulation (Supplementary Figure 1A). These results further suggest that the loss of miR-106b expression can contribute to the expression of angiogenic proteins that promote neovascularization.

### miR-106b influences HRMEC migration and choroidal vascular sprouting

We sought to characterize the effects of miR-106b on cellular processes involved in angiogenesis. We first evaluated the role of miR-106b on HRMEC migration in a wound healing/cell migration assay performed by electric cell-substrate impedance sensing (ECIS) assay. Cells were infected with LV.miR-106b, LV.shVEGFA, LV.shPERK or control LV.shGFP for 72 hours, plated at confluence and submitted to an electric pulse in the center of the well to provoke cell detachment, resulting in a 250μm diameter region devoid of cells (Figure 3E). Cell migration was quantified at 8 hours by measurement of impedance, which increases as cells repopulate the empty space. We found that HRMEC migration was reduced in all three LV-treated dishes compared to control LV.shGFP (blue line) (Figure 3F–3H). Cells infected with LV.shPERK became the least migratory (Figure 3H). LV.miR-106b prevented migration of HRMECs to a similar extent as LV.shVEGFA (Figure 3F, 3G). To confirm that miR-106b reduced migration of HRMECs, we performed a scratch assay (Figure 3I). As above, significant decreases in cell migration were observed in all treatment groups when compared to controls with a ~45% reduction in LV.shVEGFA, ~50% reduction with LV.miR-106b and ~45% with LV.shPERK when compared to control LV.shGFP (100% of migration) (Figure 3I, 3J).

NV AMD is characterized by pathological neovascularization of the choriocapillaris. We therefore used *ex vivo* mouse choroid explants and assessed sprouting angiogenesis. Similar to findings reported above, we observed a ~ 70 % reduction in sprouting area with LV.miR-106b, a ~80% reduction with LV.shPERK, while LV.shVEGFA resulted in a reduction of ~60% compared to control LV.shGFP (Figure 3K, 3L). Taken together, these results further highlight the anti-angiogenic properties of miR-106b and provide rationale to test miR-106b delivery *in vivo*.

### Intraocular injection of LV.miR-106b decreases choroidal and retinal neovascularization

**Figure 4:**
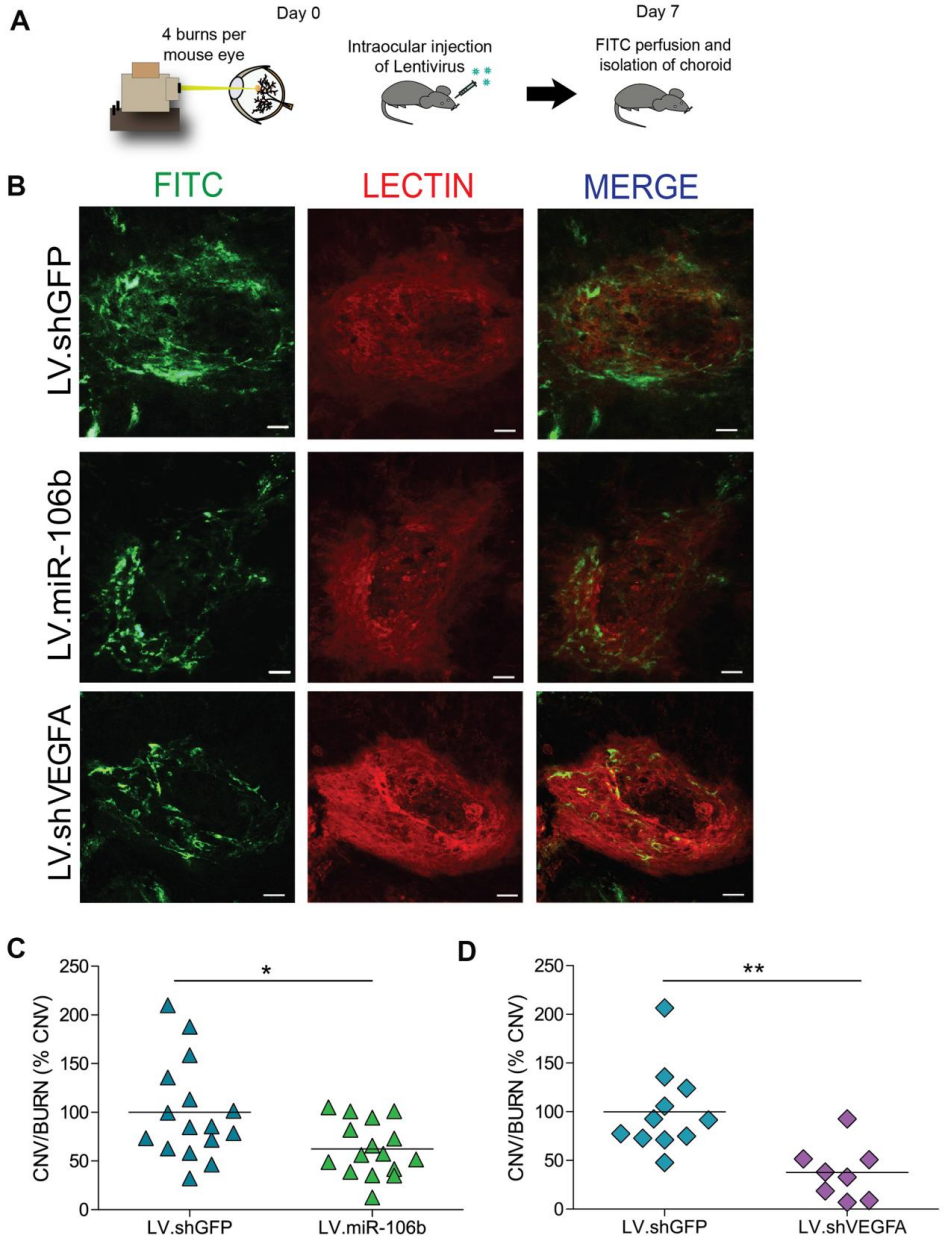
**miR-106b decreases choroidal neovascularization in a CNV mouse model.** (**A**) Schematic of intraocular injection of LV.miR-106b or LV.shVEGFA in laser burn mouse model. (**B**) Burn in red (lectin), CNV in green (FITC) and merge with LV.miR-106b or LV.shVEGFA compared with LV.shGFP. (**C**) CNV/Burns ratios quantification after LV.miR-106b treatment (n=16 burns/4 mice) and (**D**) CNV/Burns ratios quantification after LV.shVEGFA treatment (n=12 burns/3 mice). Scale bar = 50μm. Data are expressed as mean ± S.E.M. Unpaired Two-tailed Student’s t-test were used *P <0.05; **P<0.001.

Endothelial cell migration and sprouting are key processes involved in angiogenesis. We next tested the outcome of therapeutic delivery of miR-106b in models of pathological retinal angiogenesis. We first performed laser burns on 8 week-old mice to trigger CNV, directly followed by intravitreal injection of either LV.miR-106b or positive control LV.shVEGF or negative control LV.shGFP. Choroids were collected 7 days after laser burns and quantified (Figure 4A). LV.miR-106b led to ~45% reduction of neo-angiogenesis and prevented CNV to a similar extent as LV.shVEGF (Figure 4B–4D).

**Figure 5:**
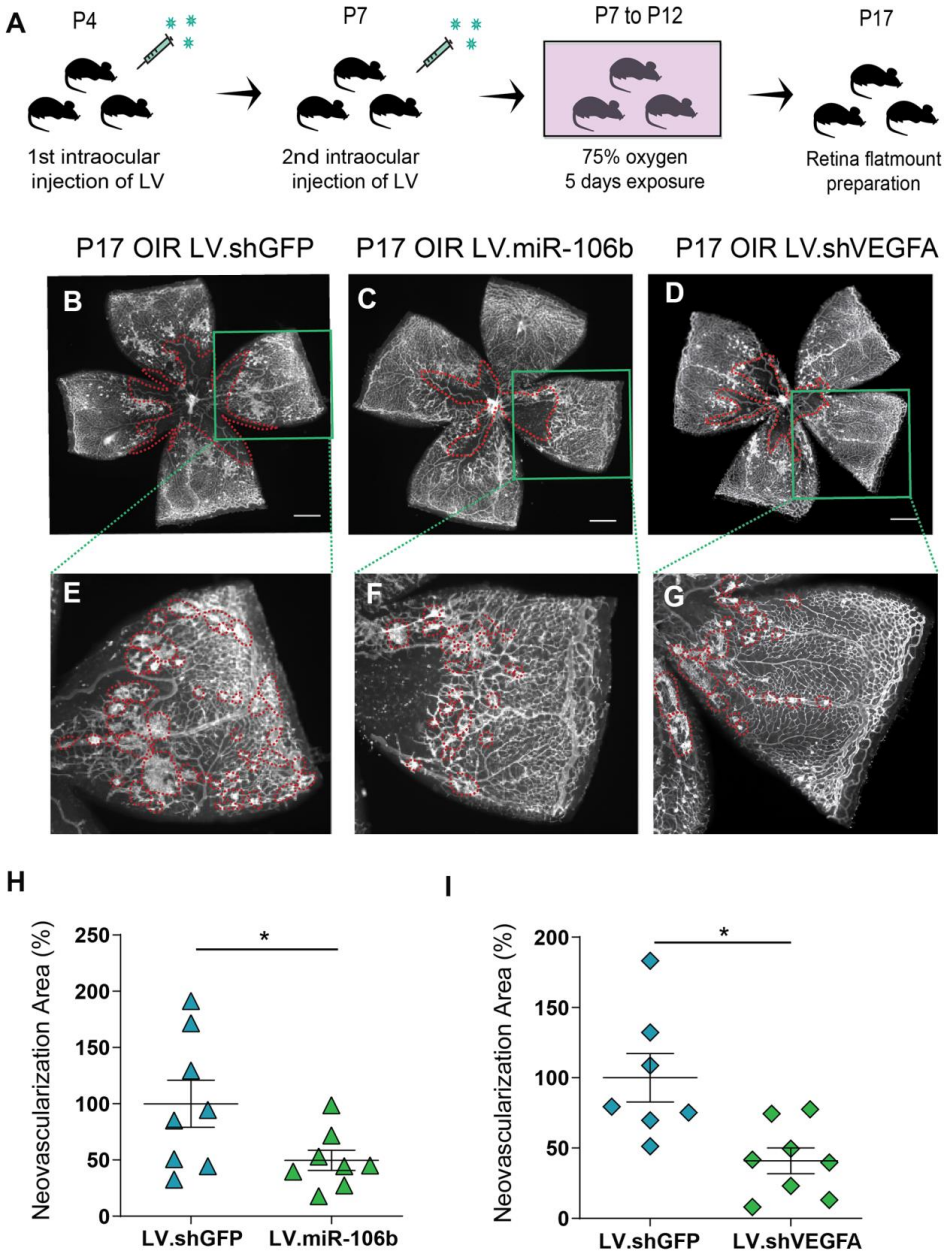
**miR-106b decreases retinal neovascularization in the OIR mouse model.** (**A**) Schematic of intraocular injection of LV.miR-106b or LV.shVEGFA in the OIR mouse model. P17 retinas were flatmounted after exposure to 75% O_2_, having received intraocular injection of LV.shGFP (**B**), LV.miR-106b (**C**), or LV.shVEGFA (**D**) and magnified (**E**–**G**). Neovascular area quantification (**H**) after LV.miR-106b injection (n=8), and (**I**) LV.shVEGFA injection (n=8). Scale bars, 500 μm (**B**–**D**) and 200 μm (**E**–**G**). Data are expressed as mean ± S.E.M. Unpaired Two-tailed Student’s t-tests were used. *P <0.05.

**Figure 6:**
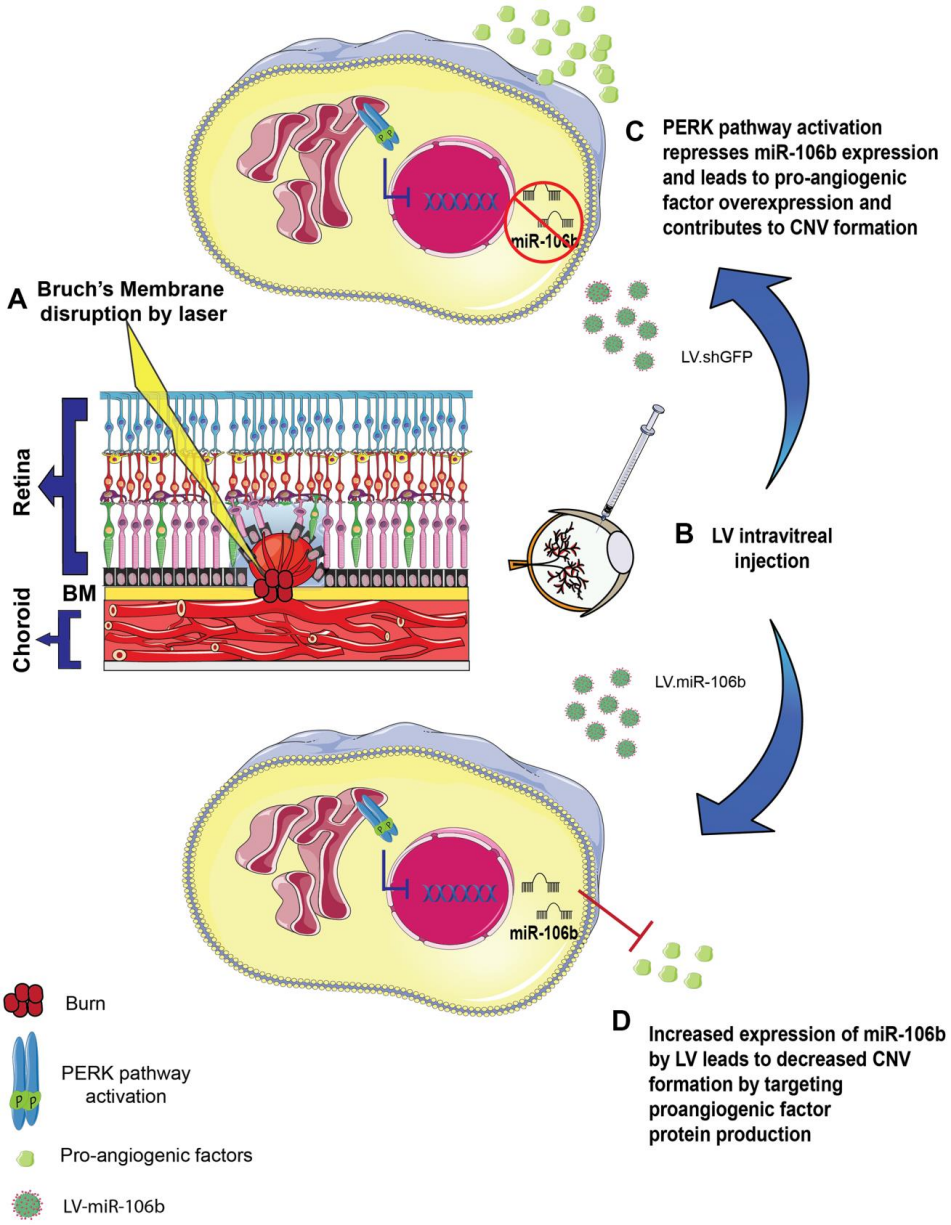
**Schematic of miR-106b impact on CNV in mouse laser burn model.** (**A**) Bruch's membrane disruption following laser burn. (**B**) Intravitreal injection of LV.shGFP or LV.miR-106b. (**C**) PERK activation represses miR-106b expression and leads to pro-angiogenic factor overexpression, contributing to CNV formation. (**D**) Increased expression of miR-106b by LV leads to decreased CNV formation by targeting proangiogenic factor protein production. (CNV: choroidal neovascularization, LV: lentivirus).

We subsequently assessed the anti-angiogenic properties of miR-106b in the mouse model of oxygen-induced retinopathy [35]. Mouse pups were injected at P4 and P7 with LV.miR-106b, negative control LV.shGFP or positive control LV.shVEGFA. From P7 to P12 pups were exposed to 75% oxygen and returned to room air from P12 until maximal neovascularization at P17 (Figure 5A). Similar to what was observed for CNV, retinas treated with LV.miR-106b showed a significant ~50% reduction in pathological neovascularization compared to LV.shGFP and a similar reduction to levels observed with LV.shVEGFA (Figure 5B–5I). Collectively, these results suggest that therapeutic delivery of miR-106b prevents pathological retinal angiogenesis (Figure 6).

## DISCUSSION

The diagnostic and therapeutic potential of miRNAs for ocular diseases is promising yet still requires proof of concept. We have previously shown that levels of miR-106b decrease in the vitreous and plasma of human patients with NV AMD [25]. In the current study, we demonstrate that much like in patients, there is a reduction in choroidal miR-106b in mouse retinas following laser burn induced-CNV. Furthermore, we provide evidence that the downregulation involves transcriptional inhibition of the 106b~25 cluster by the PERK pathway. Consequently, therapeutic intravitreal administration of miR-106b via lentiviral vectors inhibited pathological retinal neovascularization across models. These findings were supported by *in vitro* and *ex vivo* data that confirmed the role of miR-106b in preventing cellular mechanisms that lead to angiogenesis including cell migration and sprouting.

The pathogenesis of AMD is associated with oxidative stress, hypoxia, inflammation and proteotoxic stress, which can trigger pathways of ER-stress [36]. We observed activation of the PERK axis with phosphorylation of eIF2α and increase in ATF4 mRNA after laser burn. Activated PERK typically influences protein synthesis through phosphorylation of eIF2α, leading to global translation arrest [37] while transcripts with alternative upstream open reading frames, such as ATF4, are translated and can mediate an antioxidant response and expand the ER’s folding capacity [38]. With sustained activation, ATF4 can also drive transcription of pro-apoptotic factors and lead to apoptosis by inducing CHOP [38]. The PERK pathway is thought to be influenced by miRNAs. For example, miR-204 represses PERK [39] while miR-30b-5p and miR-30c-5p regulate eiF2α, and miR-214 downregulates ATF4 expression [40]. Similarly, to what has been reported in mouse embryonic fibroblasts [34], our data suggest that PERK suppresses miR-106b during choroidal neovascularization and thus renders the retina more conducive to neovascularization.

We demonstrated the anti-angiogenic properties of miR-106b in models of retinal (OIR) and choroidal neovascularization (laser CNV). The magnitude of suppression was comparable to that of suppression of VEGFA or PERK inhibition by shRNA. MiR-106b, a member of miR-17 family with reported anti-angiogenic properties [41, 42], also significantly decreased choroidal sprouting. Our results are consistent with previous studies demonstrating the anti-angiogenic properties of miR-106b in cell culture via STAT3 inhibition [43] and in mice in a hind limb ischemia model.

Currently, there are efforts to devise therapeutics that simultaneously inhibit several factors involved in retinal vascular disease given the clinical success of compounds such as Aflibercept [44]. miRNAs regulate translation of multiple genes and hence may be considered as multi-target inhibitors. Their potential to mitigate retinal disease will grow as comprehensive landscapes of miRNAs in health and disease are established [26, 45]. Preclinical studies are underway for mimics or inhibition of specific miRNAs [46]. Overall, this study demonstrates a new role for miR-106b and highlights its potential for suppressing pathological retinal neovascularization.

## MATERIALS AND METHODS

### Animals

All studies were performed according to the Association for Research in Vision and Ophthalmology (ARVO) Statement for the Use of Animals in Ophthalmic and Vision Research and were approved by the Animal Care Committee of the University of Montreal in agreement with the guidelines established by the Canadian Council on Animal Care. C57Bl/6 wild-type were purchased from Jackson Laboratory and CD1 nursing mothers from Charles River Laboratory.

### Cell line

Human retinal microvascular endothelial cells (HRMECs) (Cell System, Kirkland, USA) were used from passages 6 to 11. HRMECs were cultured in EGM-2 microvascular medium (Lonza, Switzerland). For scratch assay experiments and for Electric Cell-substrate Impedance Sensing (ECIS), cells were starved overnight then cultured in EBM-2 medium (2% fetal bovine serum).

### O_2_-induced retinopathy (OIR)

Mouse pups (C57Bl/6, Jackson Labs) and their fostering mothers (CD1, Charles River) were exposed to 75% O2 from postnatal day 7 (P7) until day 12 and returned to room air. This model serves as a proxy to human ocular neovascular diseases such as ROP and diabetic retinopathy characterized by a late phase of destructive pathological angiogenesis [47, 48]. Upon return to room air, hypoxia-driven neovascularization (NV) develops from P14 onwards [35]. Dissected retinas were flatmounted and incubated overnight with fluoresceinated isolectin B4 (1:100) in 1mM CaCl2 to determine extent of avascular area or neovascularization area at P17 using ImageJ and the SWIFT-NV method [49].

### *In vivo* imaging following laser-induced choroidal neovascularization (CNV)

*In vivo* imaging was performed using a scanning laser ophthalmoscope (Micron IV; Phoenix Laboratories, Pleasanton, CA, USA). Mice of 9 to 11 weeks of age were subjected to pupil dilation (Mydriacyl; Alcon, Mississauga, ON, Canada) and anesthetized with a mix of 10% ketamine and 4% xylazine (10μl/g body weight). Fluorescein (Alcon, 1 unit/g body weight of a 5% fluorescein dilution in 0.9% sodium chloride) was injected subcutaneously and corneas were lubricated with Optixcare ophthalmic gel (Aventix Animal Health, Burlington, ON, Canada). After a fluorescein circulation of 5 minutes, retinas were imaged before and after inducing choroidal neovascularization with 4 distinct laser burns (50μm, 300mW, 0.05s). Animals were followed-up 3, 7 and 14 days after laser burn.

### CNV induction and neovascularization labeling by perfusion with FITC

For lentiviral treatment mice of 9 to 11 weeks of age were intraocularly injected with lentivirus and their Bruch’s membranes were ruptured using an argon laser as described previously [32]. At day 3, 7 and 14 after CNV induction, mice were injected with 0.5 ml of 15 mg/ml of fluorescein isothiocyanate (FITC)–dextran (average molecular weight 20,000) (Sigma Aldrich, CA) and euthanized.

### Immunohistofluorescence

Eyes were fixed for 30 min in 4% PFA at room temperature before dissection to isolate retinas (OIR) or choroids (LB-CNV). Flatmounted retinas or choroids were stained with Rhodamine labeled Griffonia (Bandeiraea) Simplicifolia Lectin I (RL-1102; Vector Laboratories) in 1 mM CaCl2 in PBS. The sclera–choroid–RPE cell complex was mounted onto a slide, and the burns photographed with an Olympus FV1000 microscope.

### Neovascularization quantifications

Retinal neovascularization (OIR model): For visualization of pan-retinal vasculature, dissected retinas were flatmounted and incubated overnight with rhodamine-labeled Griffonia (Bandeiraea) Simplicifolia Lectin I (Vector Laboratories Inc.) in 1 mM CaCl2 in PBS for retinal vasculature. The extent of avascular area or neovascularization area at P17 was determined using ImageJ and the SWIFT_NV method [49].

Choroidal neovascularization (laser burn model): The neovascularization was captured in a Z-stack, and the lesion caused by the laser impact was captured in a single-plane image. The Z-stacks were compressed into one image, and the FITC–dextran-labeled neovascular area and the area of the lesion were measured per lesion in ImageJ.

### Western blot analysis

For assessment of choroidal protein levels, eyes were enucleated from mice 3 days after burn. RIPA buffer with anti-protease and anti-phosphatase (BioRad) was freshly prepared to manually with a piston to homogenize tissues and for cells lysis. Protein concentration was assessed by BCA assay (Sigma-Aldrich, Oakville, CA), and 30μg of protein analyzed for each condition by standard SDS-PAGE technique using Bis-Acrylamide gel 10% or 12.5% depending of protein size. Total protein transfer on nitrocellulose or PVDF membranes (Bio-Rad, Mississauga, ON, CA) was evaluated with Ponceau Red (Sigma-Aldrich, Oakville, CA). Antibody solutions and dilutions were prepared as per manufacturers’ recommendations.

### Antibodies

Phospho-eIF2α (Ser51) (Cell signaling Technology, Whitby, CA), Total-eIF2α (Cell signaling Technology, Whitby, CA), β-actin (8H10D10) (Cell signaling Technology, Whitby, CA, VEGFA (C1) (Santa Cruz Biotechnology, INC, Texas, USA), HIF1α (H1alpha67) (Novus Biologicals, Oakville, CA).

### Quantitative real time polymerase chain reaction analysis

RNA extraction was performed with TRIzol® Reagent (Life Technology, Waltham, USA) as suggested by manufacturer protocol. DNase digestion was then performed to prevent amplification of genomic DNA (Invitrogen, Waltham, USA). iScript™ Reverse Transcription Supermix for RT-qPCR (Bio-Rad, Mississauga, CA) was used to generate cDNA from 1μg of total RNA. Real time qPCR was performed to quantify gene expression using SYBR® Green reagent (Applied Biosystem TM, USA) and was processed with an ABI 7500 Real-Time PCR machine. β-actin was used as a reference gene. Primer sequences (Integrated DNA Technologies) are listed in Supplementary Table 1. miRNA extraction was performed with TRIzol® Reagent, Retrotransciption reaction was done with TaqMan MicroRNA Reverse Transcription kit (Applied Biosystem, USA) using 100ng of total RNA in each reaction following manufacturer protocol. Real-time PCR was processed with TaqMan miR assay 20X and Universal master mix II No-UNG 2x for TaqMan Reaction (Applied Biosystem, USA). Primers are listed in the Supplementary Table 1.

### Lentivirus plasmid constructions

Lentiviral constructs were produced with the PCR insertion kit (Q5 Site-Directed Mutagenesis kit, New England BioLabs®inc). The following sequence for shVEGFA and mature miR-106b sequence were inserted shVEGFA: 5’ GAGCGGAGAAAGCATTTG TTTCTCGAGAAACAAATGCTTTCTCCGCTCTTTT 3’, miR-106b: 5’TAAAGTGCT GACAGTGCAGATCTCGAGATCTGCACTGTCAGCACTTTATTTT-3’. All constructs were verified by Genome Quebec sequencing. Constructs of shIRE1α and shPERK were previously published by our group [50].

### Preparation of lentivirus

We produced infectious lentiviral vectors by transfecting lentivector and packaging vectors into HEK293T cells (Invitrogen) as previously described (Dull et al. Journal of Virology, 1998). Viral supernatants were concentrated by ultracentrifugation (>500-fold). Viral efficiency was confirmed by real-time-PCR and Western blot.

### Intravitreal injections

For the OIR model, P4, P7, C57BL/6 pups were anesthetized with 3.0% isoflurane and injected in the vitreous chamber with 0.5 μl of lentivirus. Retinas were collected at P17 for vasculature analysis. For the laser burn model, 8 to 10 week old C57BL/6 mice were injected following laser burn in the vitreous chamber with 1 μl of lentivirus. Choroids were collected 7 days post burns for CNV quantification.

### Scratch assay

Scratch assays were performed with pre-infected HRMECs cells (72hr) in 6 well plates until confluency was reached. Scratches were done with 200μl sterile tips and culture media was replaced with with EBM-2 medium (2% fetal bovine serum). Pictures were taken at time 0 (moment of the scratch) and after 8 hours with a 2x objective using an inverted microscope (Zeiss Axio Imager) and migration distances were quantified with Image J software.

### Electric cell-substrate impedance sensing migration assay (ECIS)

Real time analysis of trans and inter-endothelial impedance was performed by plating 1x 10^5^ pre-infected (72 hours) HRMECs cells into 8 well arrays (8W10E for migration assays, 40 electrodes per well) (Applied BioPhysics, Troy, NY, USA). Cells were plated at confluency and submitted to an electric pulse in the center of each well, causing localized cell detachment, resulting in a 250μm diameter devoid of cells. Cell migration was quantified by measurement of impedence, which increases as cells repopulate the empty space. The results were then normalized to the vehicle control and expressed as relative resistance. Graphical representation depicts mean and S.E.M., and light blue zones highlight time points where statistically significant differences are observed (student’s t-test, P<0.05).

### Choroid *ex vivo* explant assay

Adult C57Bl/6 mice were euthanized, and eyes were immediately enucleated and kept in ice-cold EBM basal medium (Lonza) before dissection. Choroid explants were placed in growth factor-reduced Matrigel (Corning) seeded in 24 well plates, and incubated at 37° C for 10 minutes to allow the Matrigel to solidify. 500 μL of medium was then added to each well and incubated at 37° C with 5% CO^2^ for 24 hours before lentiviral infections. Explant pictures were taken after 48 hours (at the beginning of choroid vessel growth), and at 72 hours to 96 hours post-infection to follow vessel growth. Phase contrast photos of individual explants were captured with a ZEISS Axio Oberver.Z1 microscope. Sprouting area quantification was performed using the semi-automated macro plug-in to the Image J software designed for this purpose [51].

### Statistical analyses

Data are presented as mean ± S.E.M. GraphPad Prism (GraphPad Software, San Diego, CA) was used to perform statistical analyses. We used Student’s t test to compare groups of two, and one-way ANOVA with Bonferroni post-hoc analysis for groups of 3 and more; data with P < 0.05 were considered statistically different: * denotes P < 0.05, ** P < 0.01, and *** P < 0.001.

## Supplementary Material

Supplementary Figure 1

Supplementary Table 1
