# Supplementary material for: miR-106b suppresses pathological retinal angiogenesis
Source: Aging (Albany NY). 2020 Dec 23;12(24):24836–52. doi: 10.18632/aging.202404 (PMC7803573; doi:10.18632/aging.202404)
Supplement: Supplementary Figure 1 [file aging-12-202404-s001.pdf]

SUPPLEMENTARY FIGURE

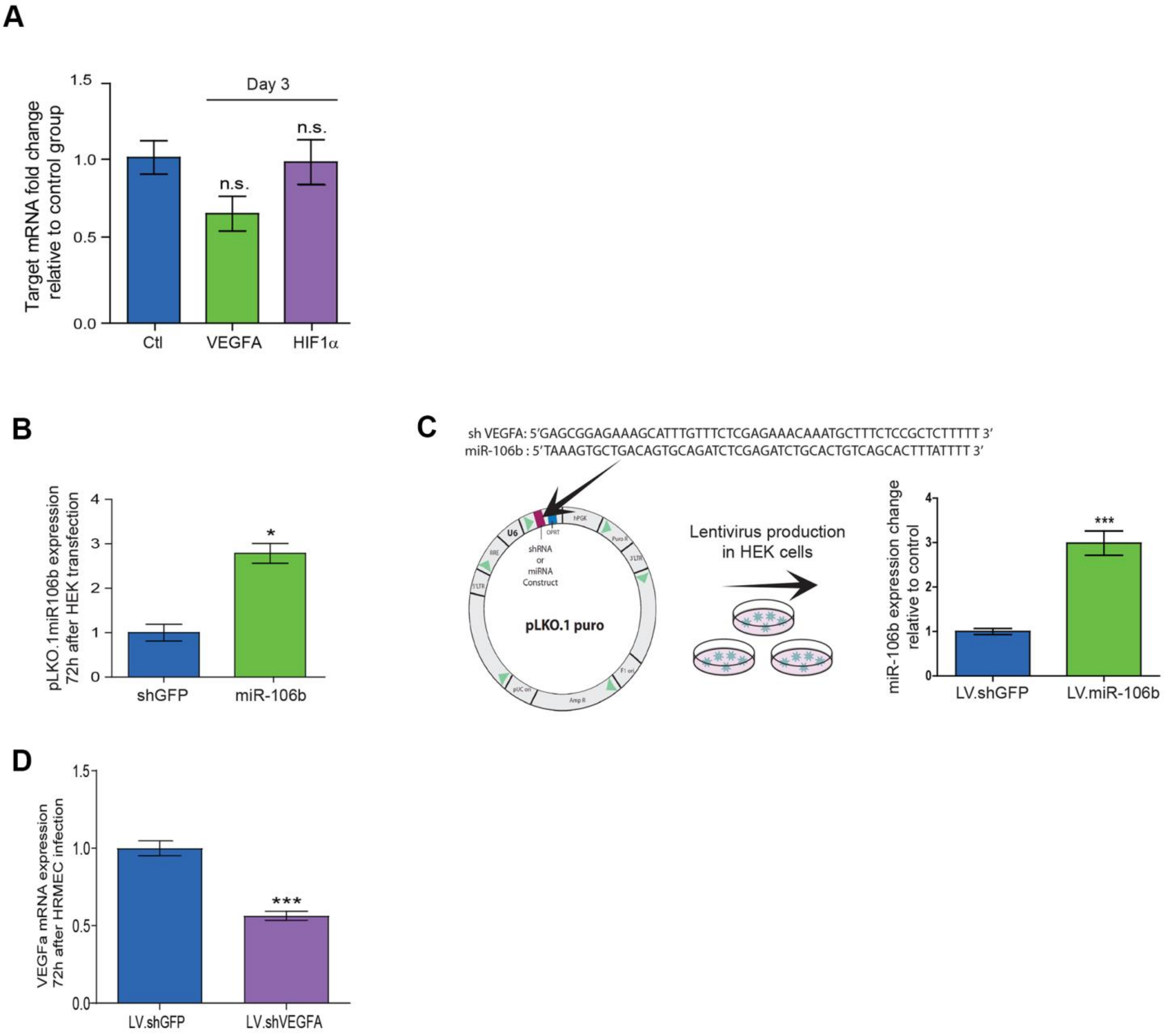

Supplemental Figure 1. (**A**) VEGFA and HIF1α mRNA expression in choroids 3 days after laser burn (VEGFA n=5, HIF1α n=7). (**B**) LV.miR-106b expression after plasmid transfection in HEK cells (n=4). (**C**) Schematic of plasmid construction, virus production and LV.miR-106b expression 72hrs after viral infection of HRMECs. (**D**) VEGFA mRNA expression in HRMECs 72hrs after LV.shVEGFA infection. Data are expressed as mean ± S.E.M. Unpaired Two-tailed Student's t-test was used for the analysis of groups of 2, and one-way ANOVA with Bonferroni post-hoc test was performed on groups of 3 or more, *P <0.05; **P<0.001; ***P<0.0001.
