# Supplementary material for: miR-106b suppresses pathological retinal angiogenesis
Source: Aging (Albany NY). 2020 Dec 23;12(24):24836–52. doi: 10.18632/aging.202404 (PMC7803573; doi:10.18632/aging.202404)
Supplement: Supplementary Table 1 [file aging-12-202404-s002.pdf]

SUPPLEMENTARY TABLE

Supplementary Table 1. List of human and mouse primers.

Genes (human)	Foward primers	Reverse primers
B-ACTIN (ACTB)	5' GTCATTCCAAATATGAGATGCGT 5'	5' TGTGGACTTGGGAGAGGACT 3'
VEGFA	5' CTCTACCTCCACCATGCCAAG 3'	5' AGACATCCATGAACTTCACCACTTC 3'
**Genes (mouse)**	**Foward primers**	**Reverse primers**
B-ACTIN (ACTB)	5' GACGGCCAGGTCATCACTATTG 3'	5' CCACAGGATTCCATACCCAAG 3'
VEGFA	5' GCCCTGAGTCAAGAGGACAG 3'	5' CTCCTAGGCCCCTCAGAAGT 3'
MCM7	5' ATGGCGCTTAAGGACTACG 3'	5'ATCCAGGTCCACATACAGTG 3'
ATF4	5' CTACTAGGTACCGCCAGAAG 3'	5' GCCTTACGGACCTCTTCTAT 3'
HIF1α	5' CGAGAACGAGAAGAAAAAGATGAG 3'	5' AAGCCATCTAGGGCTTTCAG 3'

RT-qPCR primer sequences to quantify mRNA expression in choroids (mouse) or HRMECs (human).
